# Fatty Acid Metabolism in Myeloid-Derived Suppressor Cells and Tumor-Associated Macrophages: Key Factor in Cancer Immune Evasion

**DOI:** 10.3390/cancers14010250

**Published:** 2022-01-04

**Authors:** Sophiya Siddiqui, Rainer Glauben

**Affiliations:** Medical Department of Gastroenterology, Infectious Diseases and Rheumatology, Charité—Universitätsmedizin Berlin, 12203 Berlin, Germany; sophiya.siddiqui@charite.de

**Keywords:** tumor microenvironment (TME), metabolic reprogramming, tumor-associated macrophages (TAMs), myeloid derived suppressor cells (MDSCs), lipid droplet (LD), immunosuppression

## Abstract

**Simple Summary:**

The review article discusses metabolic changes in the tumor microenvironment (TME), which in turn influences the immune cell compartment modulating the phenotype and functionality of immune cells. The main focus is to discuss the influence of increased fatty acid content in the TME, storage of fatty acids in lipid droplet (LDs) organelles in myeloid-derived suppressor cells (MDSCs), macrophages, especially tumor-associated macrophages (TAMs) and resulting functional changes towards an immunosuppressive phenotype. Thus, defining the importance of understanding the role of LD organelles in identifying new therapeutic targets for targeting immunosuppression in cancer.

**Abstract:**

The tumor microenvironment (TME) comprises various cell types, soluble factors, viz, metabolites or cytokines, which together play in promoting tumor metastasis. Tumor infiltrating immune cells play an important role against cancer, and metabolic switching in immune cells has been shown to affect activation, differentiation, and polarization from tumor suppressive into immune suppressive phenotypes. Macrophages represent one of the major immune infiltrates into TME. Blood monocyte-derived macrophages and myeloid derived suppressor cells (MDSCs) infiltrating into the TME potentiate hostile tumor progression by polarizing into immunosuppressive tumor-associated macrophages (TAMs). Recent studies in the field of immunometabolism focus on metabolic reprogramming at the TME in polarizing tumor-associated macrophages (TAMs). Lipid droplets (LD), detected in almost every eukaryotic cell type, represent the major source for intra-cellular fatty acids. Previously, LDs were mainly described as storage sites for fatty acids. However, LDs are now recognized to play an integral role in cellular signaling and consequently in inflammation and metabolism-mediated phenotypical changes in immune cells. In recent years, the role of LD dependent metabolism in macrophage functionality and phenotype has been being investigated. In this review article, we discuss fatty acids stored in LDs, their role in modulating metabolism of tumor-infiltrating immune cells and, therefore, in shaping the cancer progression.

## 1. Introduction

Cancer biology has been studied for many decades, and various factors that define the progression of cancer have been outlined as hallmarks of cancer [1]. The profound effect of cancer cell metabolic alterations and suppressive immunity in context of tumor growth have been widely researched. Immune cells functional manipulation and takeover by cancer cells to protect the growing tumor from immune invasion has drawn focus on developing novel immunotherapeutic strategies. Some of the currently used therapeutic strategies include checkpoint blockades used to reactivate T cell-mediated killing; these include anti-programmed cell death-1 (PD-1) or anti-cytotoxic T lymphocyte antigen-4 (CTLA-4) [2]. PD-1 binds to its ligand, programmed cell death ligand-1 (PDL-1), expressed on cancer cells and causes suppression of T cell-mediated anti-tumor immunity. Treatment using PD-1 as well as PDL-1 blockade is a sought after strategy for treating various cancers including Hodgkin lymphoma, head and neck squamous cell carcinoma, non-small cell lung cancer, myelomas, and more [3]. CTLA-4 is expressed on naïve (intracellularly) as well as on activated T cells (cell surface). However, on activated T cells, it interacts with B7-1 and B7-2 molecules on anti-presenting cells (APCs) promoting clonal anergy of activated T cells. CTLA-4 blockades, thus, prevent the clonal anergic state of T cells and are tested to being effective in treatment of melanomas [2,3]. However, the current known FDA approved strategies including checkpoint inhibition or other therapeutics are in many cases insufficient for reactivation of the immune system to fight cancer [3]. This encourages the need of the hour to identify additional or more efficient strategies to target cancer progression. Among the other researched therapeutic targets are tumor-derived extracellular vesicles (t-EVs) as well as altered metabolism of immune cells. T-EVs secreted by tumor cells are recognized to actively regulate various cellular activities at the tumor site including immune responsiveness (Tao et al.) [4]. T-EVs are capable of penetrating tumor-infiltrating immune cells such as T cells, MDSCs, dendritic cells, monocytes, and macrophages, and sequentially mediating immunomodulation depending on the molecules within the t-EVs. Example, upon uptake of T-EVs carrying TGF-β, T cells attain an immunosuppressive phenotype [5]. Moreover, reports indicating the influence of TGF-β and PGE2 (Prostaglandin E2) carrying t-EVs on MDSC differentiation and immunosuppression have been reported by Xiang et al. [6]. Many such reports confirm the role of t-EVs in cancer progression and recognize them as potential alternative therapeutic targets. Moreover, the growing knowledge about the role of metabolism in immune cell function has prospered the interest of cancer immunologists in immunometabolism. Metabolic reprogramming of immune cells is being defined as the new hallmark of cancer, which changes the functionality of immune cells by controlling transcriptional and posttranscriptional events that are essential for the activation of immune cells [7]. Specific metabolic pathway alterations affect immune cell functions. For instance, a shift towards glycolysis and fatty acid synthesis makes macrophages proinflammatory. On the other hand, glycolytic metabolism appears crucial for the phenotype of interleukin 17 (IL-17) producing T helper cells (Th17) (inflammatory) as opposed to the regulatory T cells (Tregs) (anti-inflammatory) phenotype [8,9]. The role of immune cells in cancer progression and the influence of metabolism on immune cell function are the primary reason for increased research efforts in the field of immunometabolism. In this review, we discuss the role of lipid metabolism with emphasis on lipid droplet storage of fatty acids in immune cells with a focus on metabolic changes in MDSCs and macrophages in the tumor microenvironment.

## 2. Various Tumor-Derived Factors Affect the Tumor Immune Microenvironment (TIME)

Among the various factors influencing the tumor immune microenvironment (TIME), Binnewies et al. reviewed the impact of tumor genotypic and phenotypic changes that affect TIME, and defined the complex changes in the immune cell compartment of the tumor that potentially influence the therapeutic responses in patients [10]. Few well-researched oncogene mediated phenotypic changes have been identified in the immune cell compartment; for example, in *kras* (Kirsten rat sarcoma virus) mutant-induced pancreatic ductal cell adenocarcinoma (PDAC), tumor cells secreting increased levels of cytokines such as of GM-CSF, actively increasing the infiltration of immunosuppressive Gr-1^−^ CD11b^+^ myeloid cells and decreasing the T cell-mediated killing of cancer cells [10]. Recently, it has been identified that *braf* and *myc* oncogenes are responsible for the *kras*-driven PDAC and are linked to the immunomodulatory effect of KRAS, which is responsible for upregulating M2 macrophage infiltration into tumor sites and for reduced numbers of CD4^+^ and CD8^+^ lymphocytes [11]. Another report regarding oncogene-mediated immunosuppression in prostate cancer shows the role of PTEN (Phosphatase and Tensin homolog) and p53 in the increased numbers of immunosuppressive MDSCs and macrophages as well as further alterations in the overall immune cell compartment [12]. *Myc* gene expression is upregulated in various cancer types and is identified to influence the innate immune response in a p19(ARF) (p19 alternative reading frame) dependent manner [13]. Other publications, reviewing the role of oncogenes in mediating immune responses, propose therapeutic targeting of oncogenes, and therefore reactivating the anti-tumor immune reaction.

Other physiological changes in the tumor cells such as an aberrant metabolism are also reportedly involved in mediating immunomodulation [10]. Metabolic reprogramming describes a dysregulated metabolism, which occurs in ailing states such as cancer. Metabolic dysregulation and transformation are thus defined as an important hallmark of cancer [1]. Cancer cells are highly active metabolically and are constantly reprogramming the nutrient fuel utilization in and around the tumor site [14]. Consistently, the tumor niche being highly proliferative, is hypoxic due to lack of sufficient blood vessels supplying oxygen into the TME. This hypoxic environment, in turn, activates hypoxia inducible factor-1 (HIF-1α), an enzyme that represents one of the various factors responsible for encouraging amplified angiogenesis and reformed metabolism, at the TME. All these factors alter the metabolite content at the tumor site. The cells within the tumor microenvironment undergo varying amounts of stress in context to pH, oxygen levels, and metabolite production [15]. Although the levels of essential nutrients may be depleting within the TME as compared to the normal tissue niche, several metabolic entity levels are on the rise including lactate, glutamate, or free fatty acids, which can be responsible for modulating the progression of cancer as well as the corresponding immune response [7]. The TME is populated by cancer as well as non-cancer cells and the influence of metabolite content between either cell type is interlinked to one another and it is important to understand the metabolic crosstalk within these cells in order to identify novel therapeutic targets [16].

Since TME is heterogeneous in context to cell type diversity or various soluble factors (cytokines, chemokines, and metabolites) that are responsible for influencing cancer progression, modulating these factors can help promote antitumor immunity. One such possible target for promoting anti-tumor immunity, as mentioned above, is the metabolic reprogramming of the tumor itself or the infiltrating immune cells, which would lead to a reduction in immunosuppressive MDSCs, TAMs, and Tregs populations [15]. In the next section, we describe certain tumor microenvironment metabolic changes that modulate not only the tumor cell but also the tumor infiltrating immune cells function and phenotype.

## 3. Impact of Altered Metabolites on Tumor Infiltrating Immune Cells and Tumor Progression

It has been reported in the past that cancer cells utilize higher amounts of glucose [17]. However, this increased glucose utilization is strongly correlated to the increased lactate secretion by cancer cells [18]. This makes lactate one of the most prominent oncometabolites produced in the TME, with a concentration as high as 30–40 mM in regions within the growing tumor [19,20]. Lactate is synthesized by lactate dehydrogenase (LDH) and its expression is upregulated in cancer cells in response to oncogene cMYC upregulation [21]. It is responsible for promoting angiogenesis, metastasis, as well as immunosuppression. Higher levels of lactate within the tumor site have been recognized to upregulate the expression of HIF-1α, which promotes the expression levels of VEGFA responsible for increased angiogenesis [22,23]. It has been reported that cancer cells secrete as well as utilize lactate and that inhibition of LDH activity in turn inhibits proliferation, invasion, and metastasis of cancer cells. In research conducted by Hou et al., they identified knocking down the *ldha* gene using LDHA siRNA in a lung adenocarcinoma (LUAD) cell line resulting in down regulation of epithelial markers such as vimentin and snail, while upregulating E-Cadherin, indicating a role of LDHA in epithelial to mesenchymal transition (EMT) [24]. A study conducted by Le et al. shows inhibition of LDHA resulted in increased oxygen consumption, as seen in a human pancreatic cancer cell line and P493 (lymphoma cell line), respectively, in turn encouraging oxidative stress and inducing cell death [25]. Lactate influences immunosuppression by affecting immune cells, viz, macrophages and regulatory T (Tregs) or effector T cells (Teff) cells, directly [23,26,27]. Higher amounts of lactate lowers NAD^+^ for glycolysis in T cells affecting T cell effector function on the other hand it promotes activation of *arginase 1* and *vegfa* and other TAM marker genes via HIF1-α signaling in macrophages [23,27].

Retinoic acid (RA), the active form of vitamin A, is produced by the conversion of retinaldehyde in a reaction catalyzed by retinaldehyde dehydrogenase (*Raldh1*, *Raldh2*, and *Raldh3*). The expression levels of Raldh enzyme are reportedly upregulated in many cancer types one such being seen in sarcoma cells and is upregulated in response to IL-13 [28]. Devalaraja et al. also identified that the high amount of RA produced by tumor cells sequentially plays a role in the differentiation of tumor infiltrating monocytes into immunosuppressive TAMs instead of differentiation into DCs. Blocking RA receptor along with PD-1 blockade increases differentiation of monocytes into these tumor suppressive TAMs, resulting in T cell mediated anti-tumor immunity [28]. It has also been reported that RA levels at TME are fivefold higher than the surrounding tissue, which influences the complex tumor microenvironment niche and the infiltrating immune cell metabolism [29]. RA metabolism in CD8^+^ T cells influences the clonal expansion and proliferation into IFN-γ producing tumor specific cell type [29].

It has been reported that various solid tumors secrete and accumulate increased amounts of fatty acids resulting in a fatty acid rich tumor microenvironment. The genes involved in lipogenesis are reportedly upregulated in cancers ranging from prostate, colonic, ovarian, liver, lung cancer, etc. [30]. This imbalance of accumulated fatty acids and lipids within TME also results in metabolic changes in tumor infiltrating immune cells. Li Jiang et al. reported in 2018 that cancer cells have increased enzymatic activity of the enzyme fatty acid synthase (FASN), which significantly increases the amount of fatty acids in ovarian cancer. Tumor infiltrating dendritic cells (TIDCs) reportedly show increased accumulation of lipids and are linked to reduced immunostimulatory ability regarding the anti-tumor T cell response [31]. TME of pancreatic ductal adenocarcinoma is enriched with long chain/very long chain fatty acids (LCFA/VLCFA) such as, for example, glycerophospholipids, and the T cell compartment is fairly impaired in PDAC, and Manzo et al. identified the role of VLCFA uptake and metabolism in CD8^+^ T cells to influence its functional impairment. They identified that accumulation of LCFAs specifically causes metabolic exhaustion with CD8^+^ T cells. VLCFA specific acyl CoA dehydrogenase (ACADVL), responsible for the initiation of mitochondrial β-oxidation of VLCFAs and LCFAs, was downregulated and identified as a potential influence on reduced metabolic fitness and impaired functionality of T cells within the PDAC tumor site [32]. The association between obesity and an increased risk of prostate cancer related deaths has been explored for decades and it is known that lipid synthesis increases in prostate cancer [33]. The increased lipid production and dysregulated fatty acid metabolism in colorectal cancer (CRC) reviewed by Rachel Brown also indicates the adversities caused by fatty acid metabolism in cancer progression [34]. Another study conducted by Watt et al. identifies the role of CD36 mediated fatty acid uptake as well as lipogenesis in prostate cancer organoids and could show that decreased proliferation and metastasis was observed upon inhibition/deletion of CD36 [35]. TME is enriched with various types of fatty content, one such being cholesterol, and has been reported to induce the exhaustion and loss of effector activity in tumor infiltrating CD8^+^ T cells. Cholesterol has been identified to upregulate the expression of exhaustion markers such as PD-1, T cell immunoglobulin and mucin domain-containing protein 3 (TIM-3), or Lymphocyte-activation gene-3 protein (LAG-3) in CD8^+^ T cells. Increased uptake of cholesterol has also been linked to X-box binding protein-1 (XBP-1) (Endoplasmic reticulum stress sensor) activation [36]. Cheng et al. studied the influence of lipid metabolic pathway mutations in non-small cell lung carcinoma (NSCLC) tissue samples and reported that higher mutations in the lipid metabolism pathway in these cancers are associated with improved immunogenicity as seen with increased infiltration of activated memory CD4^+^ T cells, γδ T cells, or CD8^+^ T cells as well as M0 and M1 macrophages and also upregulated the inflammation mediating gene profile (e.g., IFNγ, CXCL9, and CXCL10) [37]. A study by Su et al. discusses the influence of CD36 mediated enhanced lipid uptake, accumulation, and metabolism in macrophages, which results in a differentiation into a tumor-associated macrophage (TAM)-phenotype. They also reported a direct relation between increased fatty acid oxidation and upregulation of Signal Transducer and Activator of Transcription6 (STAT6) phosphorylation, in turn promoting the immunosuppressive TAM phenotype [38]. Lin et al. reported that in gastric adenocarcinomas, a higher population of tissue resident memory T cells (Trm) is associated with better prognosis and reduced metastatic state. They identified the influence of CD36 mediated fatty acid uptake and increased mitochondrial fatty acid metabolism in promoting survival and anti-tumor activity of Trms [39].

All these reports confirm, that tumor cells show a reprogrammed metabolism, which results in altered metabolite levels available in the tumor niche. A key research area now is the influence of these metabolic changes on the activities of the immune cell compartment within the microenvironment. Hence, it is important to understand and identify various potential metabolic targets that influence tumor progression. In this review, we discuss one such target or describing the role of lipid droplet mediated fatty acid metabolism in immunosuppressive on macrophage population, respectively.

## 4. Influence of Fatty Acid Storage and Metabolism in MDSC and Macrophage Differentiation

MDSCs (myeloid-derived suppressor cells) represent one of the main groups of tumor-infiltrating immune cells. They are a heterogeneous population comprising myeloid cell progenitors as well as precursors for myeloid cells, also described as immature myeloid cells (IMCs). These IMCs are capable of differentiating into macrophages, dendritic cells, or mature granulocytes. However, immature MDSCs infiltrating within the TME are a source of immature aberrantly differentiated immunosuppressive TAMs [40]. This process is driven by various factors derived from tumor cells including metabolites, cytokines, and various growth factors [41,42,43]. HIF-1α is upregulated in MDSCs infiltrating the hypoxic tumor site, activating expression of arginase 1. This is responsible in mediating T cell suppression and supporting tumor progression [44].

Under the steady-state condition, precursors of myeloid cells mature and are differentiated into granulocytes, dendritic cells, and macrophages. These mature myeloid cells form a part of the circulating leucocytes and lose the ability for self-renewal overtime. On the contrary, as mentioned above, aberrant differentiation of myeloid cells resulting in the generation of MDSCs, which are immature myeloid cells (IMCs) and express markers different from the mature differentiated myeloid cells [45]. Typically, in tumor development, the immunosuppressive MDSCs have mainly two phenotypes, monocytic MDSCs (m-MDSCs) or polymorphonuclear MDSCs (PMN-MDSCs), and a third subtype early MDSCs (eMDSCs). M-MDSCs are more prominent than PMN-MDSCs and are capable of rapidly differentiating into tumor-associated macrophages (TAMs) [45]. Monocytic MDSCs, over time within the tumor, downregulate the expression of Lys6C while upregulating markers such as CX3CR1, F4/80, and MHCII differentiating into suppressive macrophages, viz, TAMs [45]. Meyer et al. reported the role of the inflammatory tumor microenvironment and immunosuppressive MDSCs in cancer progression as co-culturing tumor-derived MDSCs with T cells results in decreased T cell proliferation and activity [46].

**Figure 1:**
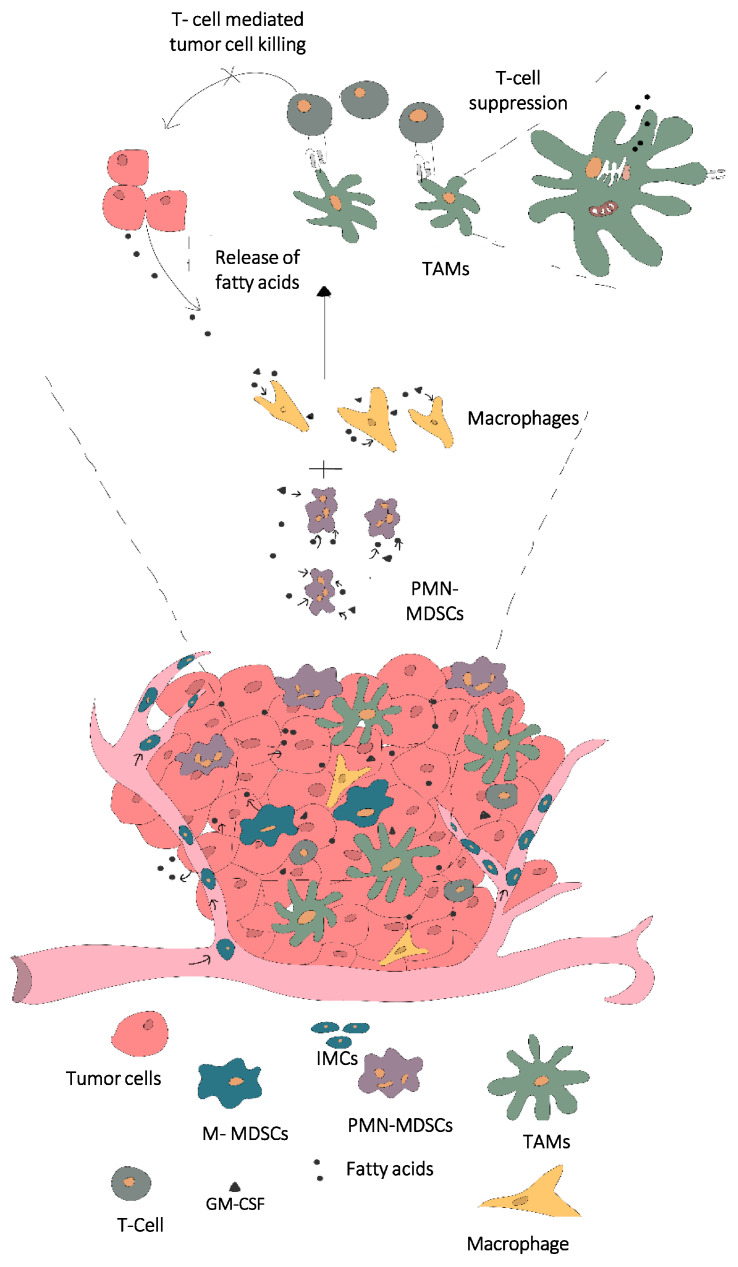
Release of fatty acids within the tumor site followed by uptake and LD-mediated storage of lipids within macrophages and MDSCs, in turn influencing the polarization and phenotype into immunosuppressive TAMs. Abbreviations: immature myeloid cells (IMCs), myeloid derived suppressor cells (MDSCs), polymorphonuclear MDSCs (PMN-MDSCs), monocytic MDSCs (M-MDSCs), tumor associated macrophages (TAMs), granulocyte macrophage colony stimulating factor (GM-CSF).

MDSCs are being recognized in recent times as potential anti-tumor therapeutic targets. With increasing understanding about the influence of intra-tumoral metabolites on MDSC differentiation, this specific effect has also become a prime focus of many researchers [43,47,48,49,50]. In the next section, we discuss the influence of, metabolism with prime emphasis on fatty acid metabolism and storage in MDSC differentiation and their role in cancer progression. Reports by Min Lee Oh et al. demonstrate that targeting glutamine metabolism in MDSCs leads to immunogenic cell death (ICD) of the tumor, decreases the recruitment and accumulation of MDSCs while increasing the number of pro-inflammatory macrophages [51]. A study conducted by Adeshakin et al. confirmed the role of lipid accumulation in immunosuppressive MDSCs. They identified the role of fatty acid transport protein 2 (FATP2) mediated lipid accumulation and increased arachidonic acid metabolism and reactive oxygen species (ROS) production in inducing higher levels of PD L1 expression in tumor cells. Thus, suggesting FATP2 as a potential anti-tumor therapeutic target [52]. Komura N et al. explored the role of tumor-derived Granulocyte colony stimulating factor (G-CSF) induced prostaglandinE2 (PGE2) producing MDSCs in driving cancer. PGE2 producing MDSCs play a role in PDL1 upregulation in ovarian cancer, also mediate suppression of CD8^+^ T cell-facilitated cancer cell killing [53]. MDSCs are identified to adjust metabolic requirements in a glucose-limited to lipid-enriched environment at the TME [48]. Xin et al. identified the role of proto-oncogene PIM-1 (a serine/threonine kinase) in Peroxisome proliferator-activated receptor-γ (PPAR-γ)-γ-mediated lipid metabolism in myeloid cells. They observed a strong correlation between PIM-1 expression, increased fatty acid oxidation, and insensitivity to immune checkpoint blockade (ICB) treatment and to PD-L1 blockade. Targeting PIM1 kinase showed reduced MDSC population at the tumor site and improved cytotoxic killing of cancer cells [54]. Other reports discuss the detailed influence of fatty acid metabolism and storage in lipid droplet organelles on myeloid cell function and differentiation. A research conducted by Wu et al. discussed how lipid droplet mediated fatty acid (oleate) metabolism in macrophages promotes an immunosuppressive phenotype [55,56]. Wu et al. also demonstrated, specifically, the storage of oleate (unsaturated fatty acid) and not stearate (saturated fatty acid) within LDs and polarization of macrophages lead to an immunosuppressive phenotype and when these polarized macrophages were co-cultured with CD 4^+^ T cell, they observed a reduced proliferative capacity of CD4^+^ T cells indicating a clear influence of oleate on immunosuppressive characterization of macrophages. They also demonstrated this immunosuppressive effect of oleate polarized macrophages to be associated with the upregulation of nitric oxide synthase and arginase-1, which is responsible for mediating T cell suppression [55,56]. Additionally, den Brok et al. reported that LDs modulate immune responses by influencing the production of eicosanoids and other inflammation mediators [57]. They also discussed the importance of LD autophagy, referred to as lipophagy, which controls the size, and number of LDs and fatty acid dependent energy generation within myeloid cells [57]. An understanding into fatty acid release from LDs by lipolysis or lipophagy is also a vast topic of discussion and involves various enzyme mediators. Key players in lipophagy are discussed in brief in Section 5 of the review. Thus, confirming the importance of understanding fatty acid storage, release, and utilization of LD-stored FAs in promoting an immunosuppressive phenotype. Figure 1 demonstrates the influence of tumor-secreted fatty acids upon uptake and storage within the tumor infiltrating macrophages, modulating them into the immunosuppressive TAM phenotype is shown in Figure 1. Another study discusses the storage of oleic acid into triglycerides in non-adipocytes therefore preventing lipotoxicity mediated disease pathogenesis or cell death [58]. These triglycerides synthesized in non-adipocytes are stored in LD organelles in leukocytes and are important players in mediating inflammation, viz, eicosanoid production or by influencing cellular signaling [59,60,61]. Compiled together this information suggests that targeting MDSC metabolism, specifically lipid droplet formation, could be a promising therapeutic target.

Apart from MDSCs, blood-derived macrophages and tissue-resident macrophages also differentiate into immunosuppressive TAMs. Macrophages are known to roughly differentiate into two main subtypes M1 macrophages (classically activated, pro-inflammatory) and M2/alternatively activated (anti-inflammatory) macrophages [62]. However, in vivo differentiation of macrophages is more complex and metabolites, cytokines, and other stimuli are capable of polarizing macrophages into a vast array of phenotypes. However, we briefly discuss the association between the role of metabolism in macrophage polarization and review important metabolic targets in MDSCs, macrophages, and TAMs within TME. It is known that macrophage metabolism varies in accordance with the functional phenotype [63]. As mentioned above, M1 and M2 macrophages have divergent metabolic requirements. Although, TAMs are not classified within the M1/M2 nomenclature of macrophage and numerous factors at the tumor site prompt their differentiation into M1-like, or M2-like, TAMs [64]. The hypoxic state at the tumor site induces activation and secretion of semaphorin3A (Sema3A) resulting in recruitment of TAMs via phosphorylation-mediated activation of vascular endothelial growth factor receptor 1 (VEGFR1), which promotes TAMs-mediated angiogenesis and immunosuppression [65]. Liu et al. identified the role of tumor microenvironmental factors in promoting a tumor-associated phenotype of macrophages [66]. They demonstrated the role of modulated glycolysis (upregulation of hexokinase-2 and other downstream glycolysis-associated enzymes: phosphofructokinase and enolase-1) in promoting the TAM phenotype. Consecutively, the observed TAM population expressed higher levels of arginase-1 and CXCR1 indicative of a suppressive phenotype [66]. Alterations in macrophage phenotype in response to altered lipid uptake have also been reported: Qin et al. observed the effect of membrane cholesterol levels on the macrophage phenotype. They demonstrated that higher membrane cholesterol levels causes changes in the F-actin (actin filament) cytoskeleton organization in a Rac1 GTPase dependent manner, causing macrophages to show higher pinocytic activity and decreased cellular migration [67], consequently, altering the macrophage functionality. As mentioned above, excess fatty acids are stored into LDs and alter the immune response. Leukocyte LDs store arachidonic acid, an essential component for the production of inflammatory mediators such as eicosanoids [57]. LD mediated storage of arachidonic acid mediates inflammation upon release [59,60]. The roles of LDs are best described for lipid storage; however, they have been implicated in a wide range of other functions, including acting as signaling platforms in lipid immobilization, vesicular trafficking, protein folding, protein storage, and autophagy [68,69]. All these reports show that LDs in mammalian immune cells, such as neutrophils and macrophages, play an important role in inflammatory or infectious processes, as increased LD accumulation also appears to be linked directly to increased type I IFN response and LD surface is also recognized as a site for Toll like receptor (TLR-7) and 9 signaling [70]. Thus, these reports indicate the importance of dysregulated fatty acid metabolism in MDSCs, macrophages, and TAMs within TME with a prime focus on LD mediated fatty acid metabolism. In the last section, we review and summarize the importance of understanding LD biology in order to find potential therapeutic targets for treating cancer.

## 5. Lipid Droplet Biology and Potential Therapeutic Targets

Lipid droplets (LD) are organelles for the storage of neutral lipids that are enveloped within a phospholipid monolayer. They are found in almost every cell type within eukaryotes and are also recognized as fatty acid storage organelles within bacteria [71,72]. LDs were long considered as inactive inert organelles, which only stored the excess fatty content of the cells [73]. However, they are now being recognized as organelles responsible for storing, as well as hydrolyzing, fatty acids, and thus affecting the levels of free fatty acids within the cell or in circulation [71]. Accumulation of LDs within cells including immune cells, hepatocytes, or adipocytes are a frequently observed phenomenon occurring in infectious and inflammatory conditions [59,60]. Excessive accumulation of lipids in non-adipocytes triggers lipid droplet formation to synthesize neutral lipids that are stored as triglycerides in LDs to protect from lipotoxicity [74]. The need of understanding LD biology and its connection with modulating cellular signalling is thus justified.

**Figure 2:**
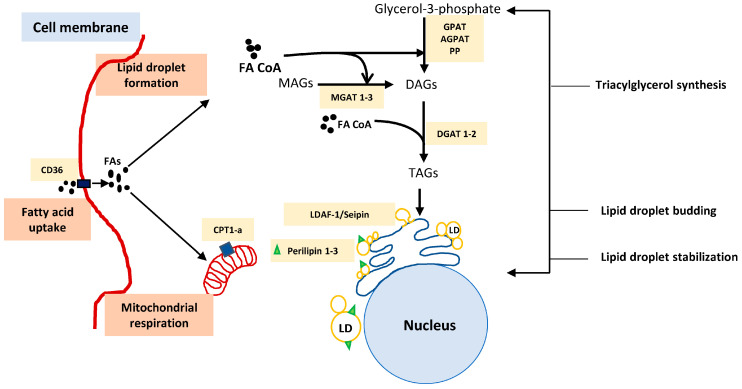
Schematic representation of the steps involved in lipid droplet biogenesis and the potential enzyme targets. Abrreviations: fatty acids (FA), fatty acyl CoA (FA CoA), carnitine palmitoyl transferase1a (CPT1-a), lipid droplet assembly factor-1 (LDAF-1), Triacylglycerol (TAGs), Diacylclycerols (DAGs), monoacylglycerols (MAGs), Glycerol phosphate transferase (GPAT), acyl CoA: Monoglycerol acyltransferase (MGAT), acylglycerol-3-phosphate-O-acyltransferase (AGPAT), lipid droplets (LD).

The composition of fatty acids within the LD as well as size, cellular location, and assembly site of LD within the cell can vary depending on various factors [75]. LDs grow ranging from 100 nm up to 100 µm in size. They may remain attached to the endoplasmic reticulum (ER) or detach and form as independent organelles [75,76]. The formation of LD is a well-orchestrated multistep process responsible for trafficking lipids including mainly sterol esters (SE), triacylglycerols (TAGs), and depending on the cell type, it may also store waxes, retinyl esters, and ethers into the synthesized droplets [76]. First step involves the synthesis of neutral lipids for LDs. The enzymes involved in synthesizing these neutral lipids are localized on the ER membrane [77]. The formation of neutral TGs begins with either of the two pathways, viz, Kennedy pathway (glycerol 3 phosphate and fatty acyl-CoA generate glycerophospholipids) or re-esterification pathway where mono- and diacylglycerols are re-esterified to generate triacylglycerols. These processes are catalyzed by glycerol phosphate acyltransferase (GPAT) and acyl-CoA: monoacylglycerol acyltransferase 1–3 (MGAT 1–3), respectively [78,79]. However, the last step of triglycerol synthesis is catalyzed by diacylglycerol acyltransferase (DGAT 1 and 2), which is responsible for catalyzing the ester bond formation between fatty acyl CoA and hydroxyl group of diacylglycerol [80,81]. The next step in LD formation, involves oil lens formation at the ER membrane and fat-storage-inducing transcript (FIT 1 and 2), an enzyme that is present on the ER membrane and is identified to play a role in initiating LD formation. FIT 1 and 2 are responsible for binding to lipids such as TAGs or SE as well as separating these lipids from ER for the LD organelle storage. Seipin and Transmembrane protein 159 (TMEM159), also known as lipid droplet assembly factor-1 (LDAF-1), form a combined machinery and are responsible for determining the exact site for LD formation on the ER [82]. The model for LD formation, after the synthesis of neutral lipids at the ER site, is a topic of extensive research and many models have been proposed to explain the detailed process of LD formation [68,76]. One such model outlying the steps involved in LD formation is demonstrated in Figure 2. As mentioned above, the synthesized LDs storing lipids can be utilized as a source of fatty acids via lipophagy. In a study conducted by Kaushik et al., it was identified that chaperone-mediated autophagy (CMA) represents a key player responsible for degrading PLIN2 and 3 on LDs, destabilizing LDs, and initiating lipolysis [83]. Followed by elevated levels of cytosolic adipose triglyceride lipase (ATGL), which further play a role in energy generation via releasing stored triglycerides [84,85], LDs having been recognized as more than just fatty acid storage organelles and potential mediators in immune responses. In this article, we reviewed the influence of LD mediated storage and metabolism of fatty acids influencing the immune response mediated by myeloid cells [57], TAMs [55], and T cells [32]. A better identification and understanding and more detailed insights into proteins and enzymes involved at the various steps of fatty acid uptake and storage into these droplet organelles and the biology of LD formation will open new doors into developing therapeutics for treating inflammatory diseases such as cancers. Next, we briefly describe the process of LD formation and the enzymes involved. LDs are inducible organelles synthesized within the cells depending on the amounts of fatty acids or synthesized TGs present. These inducible organelles, forming on the ER membrane, have a role in regulating cellular metabolism, lipid trafficking as well as cell signaling [59,60]. Understanding the various steps involved in fatty acid uptake, TG synthesis and LD formation provides insights and better understanding of the process of LDs formation and storage of triglycerides as summarized in Table 1. This will help in identifying enzymes as potential drug targets in treating fatty acid metabolism-related diseases such as diabetes, cardiovascular disorder, and cancer.

## 6. Conclusions

With new rising attention to lipid droplet organelles, various aspects of their biology are being uncovered. Numerous publications are discussing the role of altered metabolism in cancer and the importance of lipid droplets in mediating immune suppressive phenotype in MDSCs and macrophages. Moreover, recent advances have described new insights into the formation of lipid organelles and their role in immunological responses. However, many questions need to be answered. How do fatty acid storage in LD and subsequent utilization lead to immune-suppressive phenotype? What is the site of fatty acid utilization within the immune cells? How does it influence cell signaling? Since LDs in immunological cells are capable of affecting immunological responses, with increasing knowledge, and model refinement of how each step of LD formation, fatty acid storage and utilization occurs, detailed insight into the biology of this fascinating organelle will emerge, as well as new ideas on how to manipulate these hubs of metabolism for therapeutic or industrial benefits.

## Figures and Tables

**Table 1:** Summary of potential therapeutic targets involved in lipid biogenesis.

Sr. No:	Biogenesis Process for Lipids	Potential Immunomodulatory Targets in Fatty Acid Metabolism and Storage	Function in Fatty Acid Uptake Metabolism or Lipid Synthesis and Storage	Reference
1	Fatty acid uptake	CD36	Cell surface receptor involved in uptake of fatty acids into the cell	[35]
2	Fatty acid β-oxidation	CPT-1a(Carnitine palmitoyl transferase -1a)	Rate limiting step in fatty acid β oxidation, transports long chain fatty acids (e.g.: Palmitate into the mitochondria for β-oxidation)	[39,48,55]
3	TG synthesis	DGAT 1 and 2 (Diglycerol acyltransferase 1 and 2)	Involved in the final step of TG formation and catalyze ester bond between acyl CoA and hydroxyl group of diacylglycerols	[81,85,86]
		ACAT 1 and 2 (Acyl CoA:diacylglycerol transferase 1 and 2) GPAT (glycerol phosphate acyltransferase) MGAT 1–3 (Monoacylglycerol transferase 1–3)	Responsible for synthesizing sterol ester (SEs) Responsible for conversion of Glycerol3 phosphate into triglycerols Involved in the conversion of monoacylglycerols into triacylglycerols	[16,67,78,81]
4	LD lens formation	LDAF-1 (lipid droplet assembly factor -1) and Seipin	Determine the exact site of LD formation and also responsible for the transition of TGs from membrane soluble form into droplet storable forms. Deletion of LDAF-1 results in lack of LDs in low cellular levels of TGs	[82]
		FIT 1 and 2 (Fat-storage inducing transcript)	Bind to the TAGs and SEs and partition the lipids from ER membrane for transporting into LDs	[87,88]
5	LD stabilization and budding	Perilipin 1–3	Responsible for stabilizing the droplets	[89]
